# Multifunctional Electronic Skins Enable Robots to Safely and Dexterously Interact with Human

**DOI:** 10.1002/advs.202104969

**Published:** 2022-02-16

**Authors:** Guozhen Li, Shiqiang Liu, Qian Mao, Rong Zhu

**Affiliations:** ^1^ State Key Laboratory of Precision Measurement Technology and Instruments Department of Precision Instrument Tsinghua University Beijing 100084 China

**Keywords:** electronic skins, human–robot interactions, intelligent robotic control, multimodal perceptions, piezo‐thermic sensors

## Abstract

Human–robot collaboration is playing more and more important roles in current deployments of robotic systems in our lives. Haptic perception and intelligent control are essential to ensure safety and efficiency of human–robot interaction. However, existing robotic sensory and control systems are deficient in terms of performance issues, complexity, and cost. Here, the authors report a multifunctional electronic skin (e‐skin) incorporating multiple perceptions with intelligent robotic control, by which robots can safely and dexterously interact with humans. The e‐skin with a simple and cost‐effective sensory structure has multimodal perceptions of proximity, temperature, contact force, and contact position with broad measuring range, high sensitivity, and fast response. The e‐skin is applied onto robots to accomplish obstacle avoidance, safe and dexterous human–robot interaction, smart teaching, and playing Tai‐Chi, which demonstrate a broad range of applications for intelligent robots equipped with e‐skins.

## Graphical abstract

A multifunctional electronic skin (e‐skin) is proposed, enabling robots to safely and dexterously interact with human. The e‐skin with a compact hierarchical structure detects proximity, wide‐range touch force, contact position, and temperature simultaneously and independently. Robots equipped with the e‐skin combining intelligent control accomplish versatile tasks of obstacle avoidance, safe interaction, being taught by human, and playing Tai‐Chi with human.

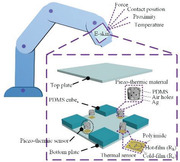

## 1 Introduction

Human–robot interactivity and safety are essential for applications of robots in our lives,^[^ ^1^, ^2^ ^]^ such as manufacturing,^[^ ^3^ ^]^ healthcare,^[^ ^4^ ^]^ and domestic services.^[^ ^5^ ^]^ These applications require robots having an ability of intelligent human–robot interaction, a function of safety control to avoid injuries to people or objects that are caused by accidental collisions^[^ ^1^, ^6^, ^7^, ^8^ ^]^ and further fulfill fine cooperation with humans. Endowing robots with the sentience of proximity and contact is the foundation for reducing collision risk and intentionally interacting with humans.^[^ ^1^ ^]^

There are two main methods to provide a robot with an ability of haptic perception, one is using proprioceptive sensors of the robot, and the other is using additional exteroceptive sensors mounted on the robot.^[^ ^9^ ^]^ The method of using proprioceptors is to monitor the robot actuator torque or current fluctuation and utilize the dynamic model of the robot to realize perceiving touch or collision.^[^ ^9^, ^10^ ^]^ However, an accurate robotic dynamic model is difficult to obtain, and the model‐based method is generally complicated and susceptible to disturbance that degrades its sensing performance.^[^ ^9^, ^10^, ^11^ ^]^

Using additional exteroceptive sensors or artificial skins mounted on robots is an alternative model‐free method. Deploying force/torque sensors at joints or end‐effectors of robots is a general approach enabling robots to perceive force. However, installing force/torque sensors at robotic joints degrades the mechanical strength of the robotic arms, and thus reduces the load capacity of the robot. In contrast, artificial skin endows a robot with sensory ability without changing its mechanical structure.^[^ ^12^ ^]^ In recent years, many tactile sensors and artificial skins based on various transduction principles have been proposed, such as capacitive,^[^ ^13^, ^14^, ^15^, ^16^ ^]^ piezoresistive,^[^ ^11^, ^17^, ^18^, ^19^, ^20^, ^21^ ^]^ thermosensitive,^[^ ^22^, ^23^, ^24^ ^]^ optic,^[^ ^25^, ^26^, ^27^, ^28^ ^]^ magnetic,^[^ ^29^, ^30^ ^]^ pneumatic,^[^ ^31^ ^]^ and triboelectric.^[^ ^32^, ^33^ ^]^ However, existing tactile sensors and electronic skins (e‐skins) used for robots suffer from the limitations of performance, complexity, and cost. For example, collision or contact forces onto robots are sometimes tiny (less than N, e.g., when a robot touches a fragile object, detecting tiny force is required so as not to crush the object^[^ ^13^ ^]^) and sometimes large (up to about hundreds of N, e.g., when a robot collides accidentally with an object during operation^[^ ^34^, ^35^ ^]^), which requires the artificial skin to have high sensitivity and wide measurement range. On the other hand, to cover on a robot, most artificial skins adopt sensor array or multisensor module matrix.^[^ ^14^, ^19^, ^20^, ^21^, ^22^, ^36^, ^37^, ^38^, ^39^ ^]^ Although these artificial skins provide robot large‐area sensing capability, a large number of sensing units leads to complex sensing array structure, complex input/output (I/O) wiring, expensive manufacturing process, complicated signal conditioning and calibration, decline in data readout rate, and large computation cost and power consumption, which obviously impedes practical applications of these artificial skins to robots.^[^ ^40^, ^41^, ^42^ ^]^ In addition, easy to mount on robots and convenient maintenance are also crucial issues for robotic artificial skins.

**Figure 1:**
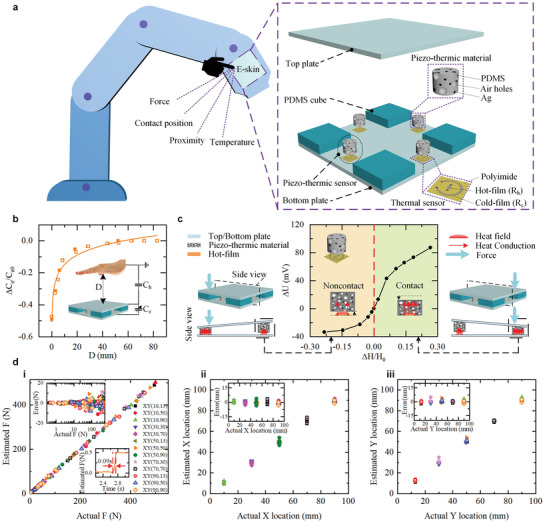
Prototype and sensing principle of the e‐skin. a) The schematic structure of the multifunctional e‐skin. b) The relative capacitance response of the e‐skin to hand approaching. *D* is denoted as the approaching distance. The inset shows the schematic diagram of the proximity sensing principle. c) The experimental response of a piezo‐thermic sensor against the shift and tilt of the top plate. Δ*H* is denoted as the displacement, where a negative Δ*H* refers to noncontact gap distance, and a positive Δ*H* (i.e., compression depth) indicates that the piezo‐thermic material contacts the thermal sensor and is compressed. *H*_0_ is the original height of the piezo‐thermic material (6 mm). d) Performance evaluation of the e‐skin for detecting contact force and contact position. d[i]) Measurement results of applied forces. The legend shows the *XY* coordinate where the force is applied. The top‐left inset shows the force errors. The bottom‐right inset shows a dynamic response of the e‐skin when putting a small weight (50 g) on the e‐skin. d[ii,iii]) Measurement results of the contact positions (*X*, *Y*). The insets show the position errors.

Here, we report a multifunction e‐skin with a simple and cost‐effective sensory structure that is easy to mount on robots. Utilizing a small number of low‐cost piezo‐thermic sensors and an integrated parallel‐plate capacitor, the e‐skin is endowed with multimodal perceptions of contact force, contact position, proximity, and temperature as shown in **Figure** **1**a. Contact force and position detections of the e‐skin are with wide measurement range, high sensitivity, and fast response, as well as immune from environment temperature variation. We also propose an intelligent robotic control strategy incorporated with multisensations of the e‐skin, which provides a robot unique ability to avoid obstacle, safely and dexterously interact with human, and be docilely taught by human. Movie S1, Supporting Information, summarizes the functions of the e‐skin and demonstrates human–robot interactions, where the robots equipped with the e‐skins nimbly elude unexpected collision to human, safely and dexterously interact with human, smartly learn skills, and play Tai‐Chi with human.

## 2 Results

The e‐skin has a simple hierarchical structure comprised of top and bottom layers as well as a middle sensing/supporting layer as illustrated in Figure 1a. The top layer is the force‐bearing plate where external forces act on. The bottom layer is the substrate of the e‐skin and also serves as a mounting plate for assembling with robots. To ensure sufficient bearing capacity, the top and bottom plates are made of steel or conductive carbon fiber. The middle sensing/supporting layer is composed of distributed piezo‐thermic sensors to detect force‐induced shift and tilt of the top plate, and distributed elastic polydimethylsiloxane (PDMS) cubes serving as elastic supporting elements for supporting the top plate. Each piezo‐thermic sensor consists of a flexible thermal sensor adhered on the bottom plate and a porous piezo‐thermic material adhered on the top plate. The thermal sensor comprises two micro platinum/chromium (Pt/Cr) ribbons fabricated on a flexible polyimide substrate. The central ribbon with a small resistance (≈60 Ω) called hot‐film (denoted as *R*_h_) is electrically heated and operated by a constant temperature difference (CTD) circuit^[^ ^43^ ^]^ (Figure S1, Supporting Information), while the circumjacent ribbon with a large resistance (≈600 Ω) called cold‐film (denoted as *R*_c_) bears low Joule heating and has almost same temperature with ambient. The piezo‐thermic material is composed of a mixture of PDMS, air holes, and silver nanoparticles (Ag). To ensure sensing performance and reduce cost, we propose a structural layout with four elastic PDMS cubes distributed at four corners of the e‐skin, and four piezo‐thermic sensors distributed at the midpoints of four edges of the e‐skin.

The multifunctional sensing principle of the e‐skin is based on piezo‐thermic transduction to detect contact force and contact position, and utilizing capacitive sensing to detect human/object approaching, as well as utilizing thermistors to detect temperature. This hybrid sensing scheme of the e‐skin avoids cross‐interference among multimodal perceptions. The piezo‐thermic transducers enable highly sensitive force and position detection using simple and low‐cost thermometry,^[^ ^44^ ^]^ which will be elaborated in a later section. The top and bottom plates of the e‐skin work as a parallel‐plate capacitor (denoted as *C*_e_) used for proximity awareness, which is based on the coupling effect of the external capacitor (denoted as *C*_h_) and the parallel‐plate capacitor *C*_e_.^[^ ^45^, ^46^ ^]^ Figure 1b illustrates the response of the e‐skin to our hand approaching. The cold‐films monolithically integrated on the piezo‐thermic sensors serve as the temperature sensors, which on the one hand detect the ambient temperature (Figure S2a, Supporting Information), and on the other hand automatically implement the temperature compensation for the piezo‐thermic sensors by the CTD scheme^[^ ^47^ ^]^ (detail in Experimental Section). Therefore, the contact force and position detections of the e‐skin are immune from environment temperature variation (Figure S2b, Supporting Information).

Based on piezo‐thermic transduction,^[^ ^44^ ^]^ the piezo‐thermic sensors detect the shift and title of the top plate by detecting the local conductive heat transfers from the hot‐films to their surrounding piezo‐thermic materials. When the e‐skin is subjected to an external force, the top plate bears the force and transmits the force to the distributed PDMS elastic supporting elements situated between the top and bottom plates. The distributed PDMS elements share the applied force and are elastically deformed, which makes the top plate to shift up and down as well as tilt correspondingly. The shift and tilt of the top plate are detected by the distributed piezo‐thermic sensors (Figure 1c). To ensure high sensitivity and broad measuring range, the piezo‐thermic sensor works in a contact‐separation mode. Specifically, when the porous piezo‐thermic material contacts the thermal sensor and is compressed by an external force, its effective thermal conductivity is increased due to the decrease of its air volume ratio, which in turn leads to a response of the hot‐film underneath the piezo‐thermic material. When the piezo‐thermic sensor works in the separation mode, the gap distance between the piezo‐thermic material and the thermal sensor can be sensitively detected by the hot‐film because of the piezo‐thermic transduction involving an air gap layer in series with a composite material. Figure 1c shows an experimental response of a piezo‐thermic sensor to the shift of the top plate, where a negative distance Δ*H* refers to noncontact gap distance and a positive distance (i.e., compression depth) indicates that the piezo‐thermic material contacts the thermal sensor and is compressed. The experimental result verifies the effectiveness of the piezo‐thermic sensor for the shift detection of the top plate. Fusing the outputs of distributed piezo‐thermic sensors enables the e‐skin to perceive the shift and tilt of the top plate that figures out the contact force and contact position. The responses of the distributed piezo‐thermic sensors under loading and unloading tests shown in Figure S3, Supporting Information, validate the effectiveness of the force/position sensing and indicate the low hysteresis of the e‐skin.

To determine the contact force *F* and contact position coordinate (*X*, *Y*) on the e‐skin from the outputs of the distributed piezo‐thermic sensors in real time, we need to establish a data‐fusion algorithm. Neural network as a machine learning method has been demonstrated to capably solve various data fusion problems.^[^ ^48^ ^]^ To ensure high accuracy and fast response, we involve the dynamic mechanism of force/position sensing into modeling and adopt a fully connected neural network to establish the dynamic model of the e‐skin (Figure S4a, Supporting Information). The time‐series data of the distributed piezo‐thermic sensors are used as the inputs of the network. The contact force *F* and contact position coordinates (*X*, *Y*) are the outputs of the network. Through optimization (Figure S4b,c, Supporting Information), the optimized neural network is structured with three hidden layers, each layer has ten neurons. To train the network and evaluate the force sensing performance of the e‐skin, we conduct a series of experiments using a force gauge, where the contact force varying from 0 to 500 N is exerted at different positions on the e‐skin. The experiment datasets (containing about 350 000 sets) used in training the neural network are different from those (containing about 90 000 sets) in evaluating the network to ensure the effectiveness of the performance validation. Figure 1d illustrates the evaluation results of the e‐skin in detecting force and contact position. The test results show that the estimated force and the estimated contact position by the e‐skin are in good agreement with the actual values. The root mean square error (RMSE) of the estimated force exerted at different positions on the e‐skin (10 × 10 cm^2^) is less than 3.2 N in the wide range of 0‐500 N (0.4 N in the low‐force range of 0‐5 N), and the RMSEs of the estimated contact position reach 1.1 mm (*X* location) and 0.9 mm (*Y* location), respectively, in the range of 0‐100 mm. The low detection limit of the force sensing is tested by putting a small weight on the e‐skin. The result (Figure 1d–i bottom‐right inset) shows the force detection limit of the e‐skin is less than 0.5 N and the response time of the e‐skin reaches 0.09 s. Movie S2, Supporting Information, shows a dynamic process using the e‐skin to measure the contact force and contact position simultaneously and display the measurement results in real time. The repeatability and durability test on the e‐skin by exerting an alternating force between 0 and 50 N for 5000 cycles is also conducted and shown in Figure S5, Supporting Information, which indicates the good stability and durability of the e‐skin.

**Figure 2:**
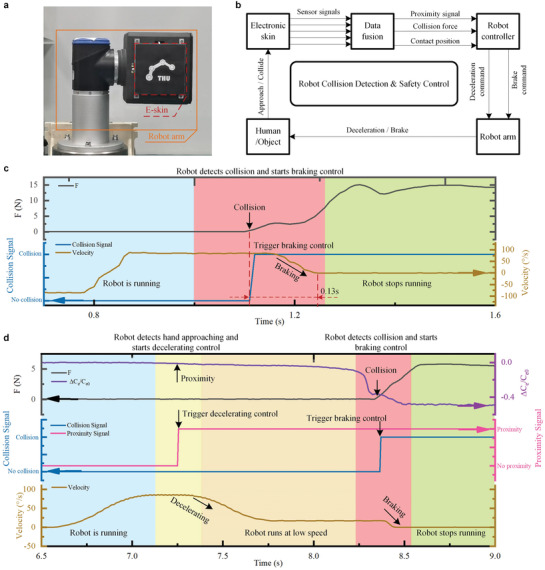
Applying the e‐skin to the robot for collision detection and safety control. a) The prototype of a robot arm equipped with an e‐skin. b) Schematic diagram of the robotic feedback control system for collision detection and safety control. c) Responses of the robot arm system when only using force detection in the safety control. d) Responses of the robot arm system when combining proximity and force detection in the safety control.

In summary, our proposed e‐skin with a compact hierarchical structure is competent to perceive proximity, temperature, contact force, and contact position with wide measuring ranges (0‐500 N, 100 cm^2^), high sensitivity (0.5 N), fast response (0.09 s), low hysteresis, and good reliability. Unlike traditional artificial skins utilizing a large number of sensing units that require complex array structure and complicated conditioning circuit, our e‐skin makes good use of only four distributed piezo‐thermic sensors to achieve fast and accurate detections of contact force and contact position on a large area, and thus takes merits of simple structure, low cost, and easy operation. The e‐skin can be further scaled up without additional sensor cost and its geometrical shape can be modified to fit various robot arms. One example of a customized e‐skin with a curved area of 139 cm^2^ mounted on a six‐degree‐of‐freedom (6‐DoF) robot arm is shown in Figure S6, Supporting Information. In addition, the e‐skin is easy to mount on a robot. **Figure** **2**a shows a homemade robot arm equipped with our e‐skin.

As mentioned above, collision detection and safety interactive control are significant for robots.^[^ ^11^, ^49^ ^]^ A smart robot should have an ability to detect obstacle and be aware of collision risk and makes fast response to minimize risk of injury. We utilize our e‐skin to execute robotic collision detection and safety control as shown in Figure 2b. The e‐skin detects human/object approaching and collision in real time and transmits the detected proximity signal and collision information (force and position) to the robot controller. When the detected signals exceed the predefined safety thresholds (|Δ*C*_e_/*C*_e0_| > 0.02 or *F* > 0.5 N), the robot triggers its safety control (deceleration or brake) in time to avoid collision injuries (Movies S3–S5, Supporting Information). Figure 2c illustrates the responses of robotic collision detection and safety control when using only collision force signal detected by the e‐skin. A human hand approaches the running robot arm (running at 85° s^−1^), then a collision happens. When the detected collision force exceeds the predefined thresholds (0.5 N), the robot triggers a braking response. The result shows that the peak collision force reaches about 15 N, and the robot arm brakes in about 0.13 s after the collision occurs. If we combine the proximity signal with the collision force in the safety control, the robot collision damage can be greatly reduced and the safety level is highly improved. Figure 2d illustrates the corresponding responses of the robot when combining the proximity signal with the force detection in the safety control policy. When the e‐skin detects an object approaching by the proximity signal, the robot actively decelerates promptly (down to a predefined velocity of 18° s^−1^), which greatly reduces the subsequent collision strength. The result shows that the peak collision force is reduced to about 6 N. The dynamic processes of collision detection and safety control of the robot utilizing collision force mode and proximity/force combination mode, respectively, are demonstrated in Movies S4 and S5, Supporting Information.

**Figure 3:**
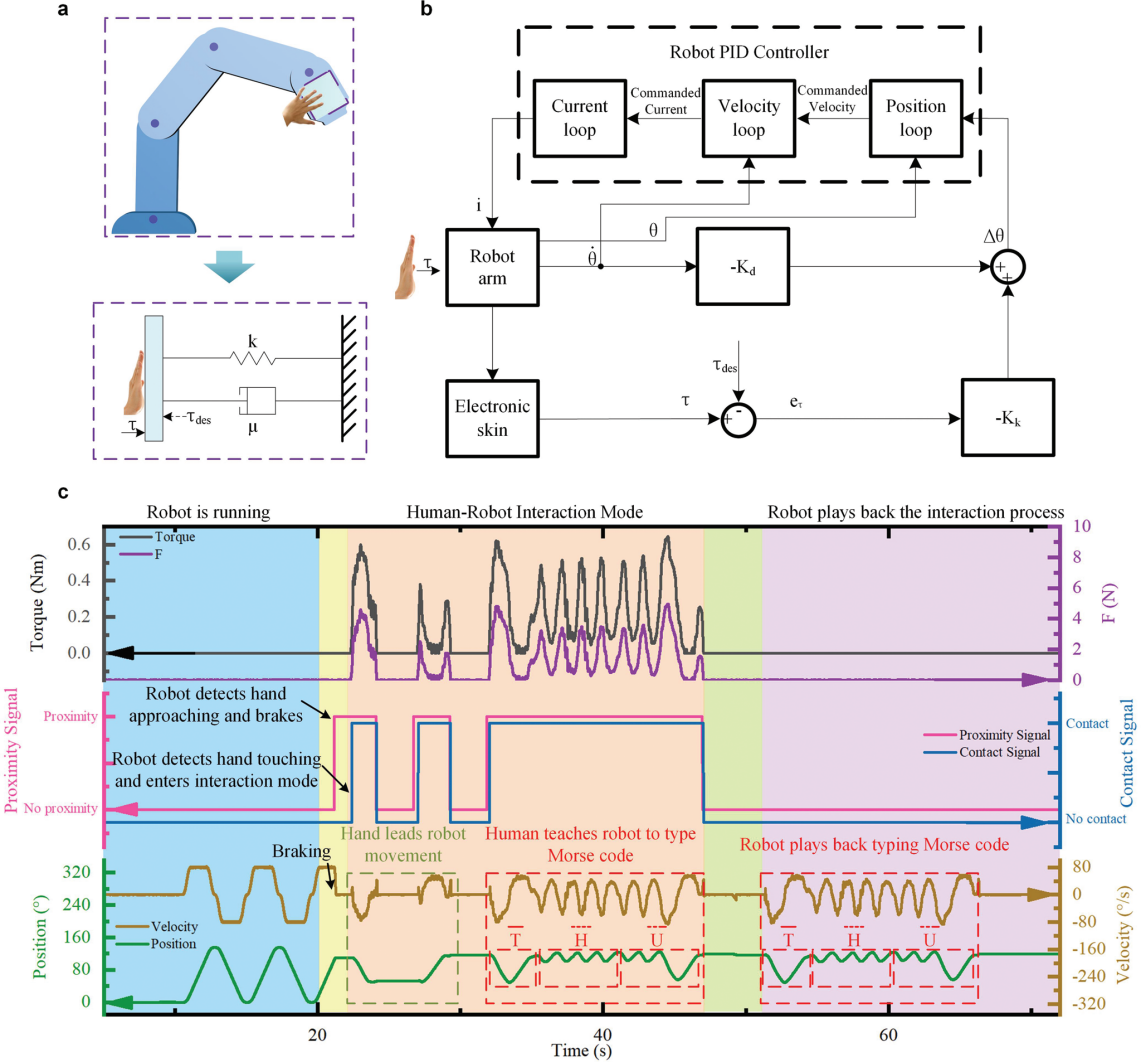
Applying the e‐skin to the robot for human–robot interaction (HRI). a) The virtual spring‐damping system of the robot arm.*τ*is the interaction torque on the robot, *τ*_des_ is a desired acting torque, *k* and *μ* are the stiffness coefficient and damping coefficient of the virtual system respectively. b) Flowchart of the control system for human–robot interaction. c) Responses of the robot arm system in the human–robot interaction and a demonstration of teaching the robot to type Morse code.

In addition to collision safety control, it is also necessary for robots to intentionally interact with human to fulfill human–robot collaboration. Safe and efficient human–robot interaction is important in human–robot coexistence circumstances. We expect to develop a harmonious relationship between human and robot, that is, both can work efficiently and independently in noninteractive mode. When an intentional interaction happens, the robot can actively comply with the human in a safe and dexterous way. To achieve this goal, we further propose an interactive control strategy that equates the robot arm as a virtual spring‐damping system to implement a safe and dexterous interaction with human as shown in **Figure** **3**a. We hypothesize that the robot arm follows a spring‐damping kinetic mechanism (detail in Experimental Section) and propose the following interactive control law:

(1) $$\Delta \theta =-K_{k}e_{\tau }-K_{d}\overset{•}{\theta }$$

where Δ*θ* is the angle increment of the robot, *K*_k_ and *K*_d_ are the coefficients related to the stiffness and damping of the kinetic system respectively, *e*_*τ*_ = *τ* − *τ*_des_ is torque deviation, *τ* is the interaction torque on the robot detected by the e‐skin, *τ*_des_ is a desired acting torque, and $\overset{•}{\theta }$ is the robot angular velocity. By using this control strategy, a human can effortlessly lead a robot arm to move forward and backward via touching on the e‐skin.

When a human hand touches on the robot arm, the interaction torque is detected by the e‐skin. And then the angle increment of the robot is determined by using Equation (1) and transmitted to the PID controller of the robot to fulfill the subsequence movement of the robot in real time (Figure 3b). Figure 3c shows the results of the human–robot interaction and a demonstration of human teaching the robot to type Morse code. When a human hand approaches the running robot arm, the robot perceives the hand timely and brakes promptly for safe entry into the interactive mode. When the human hand touches on the robot arm, the robot detects human contact and triggers human–robot interaction mode immediately. That is, the robot is controlled to keep in step with the human hand. Specifically, the hand leads the robot to move forward and backward intentionally via the e‐skin. In interactive mode, the motion of the robot arm can be well controlled by using the proposed interactive control strategy. In other words, the human can lead the robot to perform arbitrary movements by the aid of the e‐skin. As a demonstration, we teach the robot to type the Morse code of “THU,” where the motion path of the robot arm represents the symbol of Morse code. In virtue of high sensitivity and fast response of the e‐skin as well as robust interactive control, the movement of the robot is smooth and the teaching is effortless. After teaching, the robot independently and accurately replays typing Morse code of “THU” in real time as shown in Figure 3c. The dynamic process of human–robot interaction and teaching/playback is demonstrated in Movie S6, Supporting Information.

**Figure 4:**
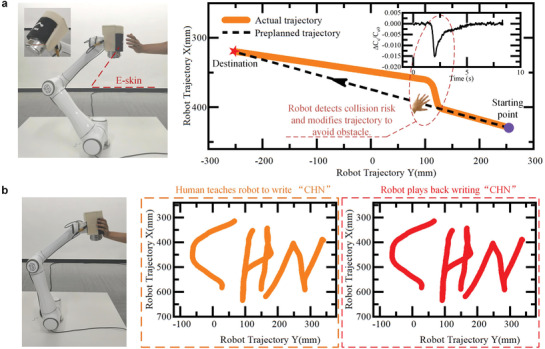
Applying e‐skin on a multi‐degree‐of‐freedom robot arm to implement human–robot interaction and teaching. a) A 6‐DoF robot arm equipped with our e‐skin (Left). Motion trajectory of the 6‐DoF robot arm to implement obstacle avoidance (Right). b) Human teaches the robot to write the word “CHN” by using hand touching on the e‐skin, and the robot accurately and independently replays writing “CHN” in real time.

Furthermore, we apply an e‐skin (area of 139 cm^2^ with a curved profile customized to fit a 6‐DOF robot arm) to a commercial 6‐DoF robot arm (detail in Experimental Section), as shown in **Figure** **4** and Figure S6a, Supporting Information. The e‐skin is mounted on the end‐effector of the robot. By using the proximity detection of the e‐skin, the 6‐DoF robot arm accomplishes obstacle avoidance, that is, timely modifies the moving trajectory of the robot, to ensure safety in case a human is close to the robot, the results are shown in Figure 4a and Movie S7, Supporting Information. In addition, the 6‐DoF robot arm can be led to move flexibly by our hand touching on the e‐skin. By this way, we can play Tai‐Chi with the robot (Movie S8, Supporting Information) or teach the robot to handwrite words (Movie S9, Supporting Information). As shown in Figure 4b, human hand teaches the robot to handwrite the characters “CHN.” After teaching, the robot can accurately and independently replay handwriting “CHN” in real time. Compared to programming‐based path planning or image‐based robotic teaching,^[^ ^50^ ^]^ hand‐by‐hand teaching enables simplifying operation processes, enhancing efficiency, and flexibility of robotic manipulation, and thus facilitating human–robot cooperation.

Above demonstrations show that safe and dexterous human–robot interactions are well accomplished by equipping our multifunctional e‐skin on robots. Using this cost‐effective e‐skin, even traditional industrial robots can be easily turned into smart collaborative robots that are able to safely and dexterously interact with humans and be docilely taught by humans. It is greatly meaningful to deploy or modify robots for intelligent manufacturing by a simple and low‐cost way.

## 3 Conclusion

In conclusion, we propose a cost‐effective multifunctional e‐skin enabling multimodal perceptions of contact force, contact position, temperature, and proximity. Utilizing only four low‐cost piezo‐thermic sensors, the e‐skin is capable of perceiving contact force and contact position, taking merits of broad measuring ranges (0‐500 N, >100 cm^2^), high sensitivity (0.5 N, 1 mm), fast response (0.09 s), low hysteresis, and good reliability. Moreover, the monolithically integrated cold‐films on the piezo‐thermic sensors eliminate the environmental temperature effects on the force/position detections of the e‐skin and also provide the temperature sensing capability for the e‐skin. Proximity detection is also integrated in the e‐skin via the parallel‐plate capacitor to provide obstacle avoidance ability for robot. The e‐skin is easy to assemble with robots, can be modified to fit various robot arms, and scaled up without additional sensor cost. We also propose an intelligent robotic control strategy incorporated with multisensations of the e‐skin to realize robotic collision/obstacle avoidance, safe and dexterous human–robot interaction, hand‐by‐hand teaching, and playing with human. Our demonstrations show that the multifunctional e‐skin facilitates human–robot collaboration and would expedite robotic applications in intelligent manufacturing, medicine, and home service.

## 4 Experimental Section

### Fabrication of the Electronic Skin

To ensure sufficient bearing capacity, the top and bottom plates (3 mm thick) were made of steel or conductive carbon fiber.

The thermal sensor was fabricated on a polyimide substrate (AP8525R, DuPont Co. Ltd.). First, a 30 µm photoresist was sprayed onto the substrate and patterned by using photolithography, then the Cr/Pt (35 nm/140 nm) films were sequentially deposited by sputtering, and patterned by a lift‐off process. Finally, a 4 µm parylene film was coated on the thermal sensor as a protective layer.

The piezo‐thermic material was fabricated by mixing silver nanoparticles (diameter < 100 nm, S110970, Aladdin Co. Ltd.), prepared PDMS solution (Sylgard 184, Dow Corning Company, the ratio of base agent: cross‐linker was 10:1 wt%), and citric acid monohydrate particles (CAM). The volume ratio of silver nanoparticles was 2.5 vol%. The mass ratio of PDMS:CAM was 1:3.5. The mixture was cured at 75 °C for 2 h in PMMA cylindrical mold (diameter 10 mm, height 6 mm) and immersed in ethanol for 24 h to dissolve the CAM to form the porous material. The fabricated piezo‐thermic material had the same size with the PMMA mold.

The PDMS cube was composed of two PDMS layers with different cross‐linker ratios. The first PDMS layer was cured with a cross‐linker ratio of 25:1 wt% in the mold (30 × 30 × 3 mm^3^) at 75 °C for 2 h, then another same mold was superimposed to cure the second PDMS layer with a cross‐linker ratio of 28:1 wt%.

Finally, the authors glued the PDMS cubes and the thermal sensors to the bottom plate, and paired the thermal sensors with piezo‐thermic materials as shown in Figure 1a; then the top plate was glued with piezo‐thermic materials and the PDMS cubes. Each thermal sensor pairing with a functional piezo‐thermic material constituted a piezo‐thermic sensor.

### Signal Conditioning of the Electronic Skin

As shown in Figure S1, Supporting Information, the capacitance of the e‐skin was detected using a commercial capacitance digital converter (CDC) (AD7150, Analog Device Co. Ltd.). The CTD circuits were used for signal conditioning of the thermal sensors of distributed piezo‐thermic sensors on the e‐skin. The two Cr/Pt films of the thermal sensor were connected to the Wheatstone bridge. The CTD of the hot‐film of a thermal sensor was achieved by the feedback circuit. All signals of the e‐skin were sampled and collected using a microcontroller unit (MCU) (STM32L476, STMicroelectronics). The signal sampling cycle of the e‐skin was 5 ms.

### Device Characterization and Experiment Measurement

Proximity detection was conducted by fixing the object or hand on a mechanized *z*‐axis stage (Handpi HLD) embedded with a displacement ruler. Temperature compensation and responses of the e‐skin were tested in a temperature‐controlled oven (OGH60, Thermo Fisher Co. Ltd.). Force and contact position measurements were conducted by applying force at different positions on the e‐skin using a mechanized *z*‐axis stage with a force gauge (Handpi HP‐1K).

The force sensitivity and measuring range of the e‐skin highly depended on the compositions of the PDMS cubes (supporting elements) and the piezo‐thermic material. Therefore the compositions of the PDMS cubes and the piezo‐thermic materials were optimized. Figure S7a, Supporting Information, was the Young's moduli of the PDMS cubes with different cross‐linker ratios, which presented that the Young's modulus of the PDMS cube could be regulated by adjusting the cross‐linker ratio. The PDMS cube composed of two PDMS layers with Young's moduli of 163 kPa (cross‐linker ratio 28:1 wt%) and 407 kPa (cross‐linker ratio 25:1 wt%), respectively, was adopted to meet both requirements on high sensitivity and broad measurement range, and the thickness of each PDMS layer was 3 mm. Furthermore, the authors optimized the composition of the piezo‐thermic materials of the e‐skin. The experimental result (Figure S7b, Supporting Information) illustrated that properly increasing the porosity and the ratio of Ag could improve the measuring range of the piezo‐thermic sensor under large force. Therefore, the porosity and the ratio of Ag were optimized to be 70 and 2.5 vol%, respectively.

To calibrate the force and position sensing of the e‐skin, a force ranging from 0 to 500 N was applied at 13 different positions of the e‐skin using a force gauge and record the corresponding signals of the e‐skin. About 60 different forces were applied at each position. The authors collected about 350 000 sets of experimental dataset to train the neural network by using MATLAB and collected another dataset containing about 90 000 sets to evaluate the sensing performance. Both the number of hidden layers and neurons were determined through optimization (Figure S4, Supporting Information). The training learning rate was set to 0.01, and the sigmoid function was used as the activation function of the neurons. The optimized network contains three hidden layers and each hidden layer had ten neurons.

An e‐skin was mounted on a homemade robot arm composed of a robot joint (RGM14, Kollmorgen Company). The multisensory signals of the e‐skin were sampled and transmitted to a PC via a cluster communication port (COM). The force and contact position calculated by the neural network were displayed on a computer interface programmed by LabVIEW, at the same time were used to the customized compliant control algorithm programmed by using TwinCAT3. The control cycle of the robot was 5 ms.

Another e‐skin was mounted on a commercial 6‐DoF robot arm (EC66, ELITE Co. Ltd). The signals of the e‐skin were sampled and transmitted to a PC via COM, and then were processed to generate control commands by using the customized compliant control algorithm programmed in Python (Python 3.8.0). Then the commands were transmitted to the commercial robot controller‐box via local area network. The control cycle of the 6‐DoF robot arm was 10 ms.

### Proximity Detection Principle of the Electronic Skin

As shown in Figure 1b, when an object or hand approached the e‐skin, the fringe electric field of the e‐skin was intercepted and shunted to the ground, which caused the capacitance of the e‐skin to decrease. The relationship between the capacitance of the e‐skin and the proximity distance could be expressed as:^[^ ^45^ ^]^

(2) $$C_{e}=\frac{4\varepsilon W}{\pi }\ln\left(\frac{2L}{D}\right)$$

where *C*_e_ was the capacitance of the e‐skin, *L* and *W* referred to the geometry length and width of the external object or hand, respectively,*ε*was a constant related to the electrical properties of the object or hand approaching, *D* was the distance between the e‐skin and the object or hand approaching. The model indicated that the e‐skin had the capability to perceive proximity. It was noted that the proximity response also depended on the electrical properties of the approaching object^[^ ^2^ ^]^ (Figure S8, Supporting Information).

### Temperature Measurement and Compensation Principle of the Thermal Sensor on the Electronic Skin

As shown in Figure S1, Supporting Information, the hot‐film and cold‐film of a thermal sensor were connected into the Wheatstone Bridge of the CTD circuit. Because the resistance of the cold‐film (≈600 Ω) was much larger than that of the hot‐film (≈60 Ω), the heating power of the cold‐film could be ignored. The cold‐film temperature and the ambient temperature were almost equal, which could be expressed as:

(3) $$R_{c}=R_{c0}\left(1+\alpha_{c}T\right)$$

where *R*_c0_ was the resistance of cold‐film at 0 °C, *α*_c_ was the temperature coefficient resistance (TCR) of cold‐films, and *T* was the ambient temperature. According to the CTD circuit, the resistance of cold‐film could also be expressed as:

(4) $$R_{c}=\frac{U_{c}}{U-U_{c}}R_{b}-R_{t}$$

Combining the Equations (3) and (4), the temperature *T* could be figured out as following:

(5) $$\lambda =\frac{U_{c}}{U-U_{c}}=\frac{\alpha_{c}R_{c0}}{R_{b}}T+\frac{R_{c0}+R_{t}}{R_{b}}$$

where $\frac{U_{c}}{U-U_{c}}$ was denoted as *λ*.

Furthermore, when the Wheatstone bridge reached a balanced state by the feedback circuit, the resistance relationship of the CTD circuit could be obtained:

(6) $$R_{a}\left[R_{t}+R_{c0}\left(1+\alpha T\right)\right]=R_{b}R_{h0}\left(1+\alpha T+\alpha \Delta T\right)$$

where Δ*T* was the temperature difference between the hot‐film and the ambient, *R*_h0_ was the resistance of hot‐film at 0 °C, and*α* was denoted uniformly as the TCRs of the cold‐film and hot‐film as they were approximately equal. Moreover, the resistance ratio of the bridge was set as:^[^ ^43^, ^47^ ^]^

(7) $$\frac{R_{a}}{R_{b}}=\frac{R_{h0}}{R_{c0}}$$

Substituting Equations (6) into (7), Δ*T* could be expressed as:

(8) $$\Delta T=\frac{R_{t}}{\alpha R_{c0}}$$

which indicated that the temperature difference between the hot‐film and the ambient was independent of the ambient temperature. The temperature differences of all thermal sensors were set as 20 °C.

### The Compliance Control Of Human–Robot Interaction for the Robot Equipped with Electronic Skin

As shown in Figure 3a, the authors equated the robot arm as a virtual spring‐damping system. When the human hand lightly touched the robot arm, the total torque of the kinematic system could be expressed as:

(9) $$\tau_{\mathrm{total}}=\tau -\tau_{\mathrm{des}}+k\Delta \theta +\mu \overset{\cdot }{\theta }$$

where *τ*_total_ was the total torque, *τ* was the interaction torque on the robot, *τ*_des_ was a set desired acting torque, $\overset{\cdot }{\theta }$ was the angular velocity of the robot arm, Δ*θ* was the angle increment of the robot arm, *k* and *μ* were the stiffness coefficient and damping coefficient of the spring‐damping system, respectively. Under the quasi‐static state, the total torque was ≈0, so the angle increment of the robot arm could be determined as:

(10) $$\Delta \theta =-K_{k}e_{\tau }-K_{d}\overset{\cdot }{\theta }$$

where *e*_*τ*_ = *τ* − *τ*_des_ was torque deviation, $K_{k}=1/k$, and $K_{d}=\mu /k$. The coefficients of *τ*_des_, *K*_k_, and *K*_d_ were set as 0.25, 3.5, and 0.012, respectively, through optimization trial to achieve compliant movements of the robot following human hand, including pushing forward and stepping backward maneuvers.

## Conflict of Interest

The authors declare no conflict of interest.

## Supporting information

Supporting Information

Supplemental Movie 1

Supplemental Movie 2

Supplemental Movie 3

Supplemental Movie 4

Supplemental Movie 5

Supplemental Movie 6

Supplemental Movie 7

Supplemental Movie 8

Supplemental Movie 9

## Data Availability

The data that support the findings of this study are available from the corresponding author upon reasonable request.
