# Supplementary material for: Multifunctional Electronic Skins Enable Robots to Safely and Dexterously Interact with Human
Source: Adv Sci (Weinh). 2022 Feb 16;9(11):2104969. doi: 10.1002/advs.202104969 (PMC9008439; doi:10.1002/advs.202104969)
Supplement: Supplementary file 1 — Supporting Information [file ADVS-9-2104969-s006.pdf]

WILEY-VCH

ADVANCED SCIENCE Open Access

Supporting Information

for *Adv. Sci.*, DOI: 10.1002/advs.202104969

Multifunctional electronic skins enable robots to safely and dexterously interact with human

*Guozhen Li#, Shiqiang Liu#, Qian Mao, and Rong Zhu**

Supporting Information

Multifunctional electronic skins enable robots to safely and dexterously interact with human

*Guozhen Li#, Shiqiang Liu#, Qian Mao, and Rong Zhu**

State Key Laboratory of Precision Measurement Technology and Instruments, Department of Precision Instrument, Tsinghua University, Beijing 100084, China

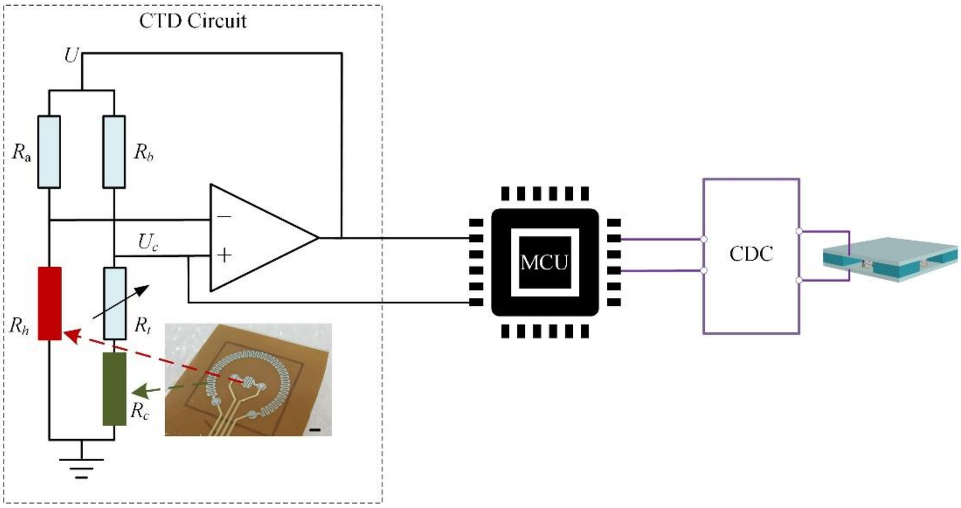

Figure S1. Schematic of the signal conditioning circuit of the e-skin. The top/bottom plates of the e-skin are connected directly to CDC for proximity detection. The CTD circuits are used to condition the signals of the thermal sensors of the distributed piezo-thermic sensors on the e-skin. The detailed configuration of the CTD circuit is presented in Methods. Scale bar in the inset of CTD circuit, 1 mm.

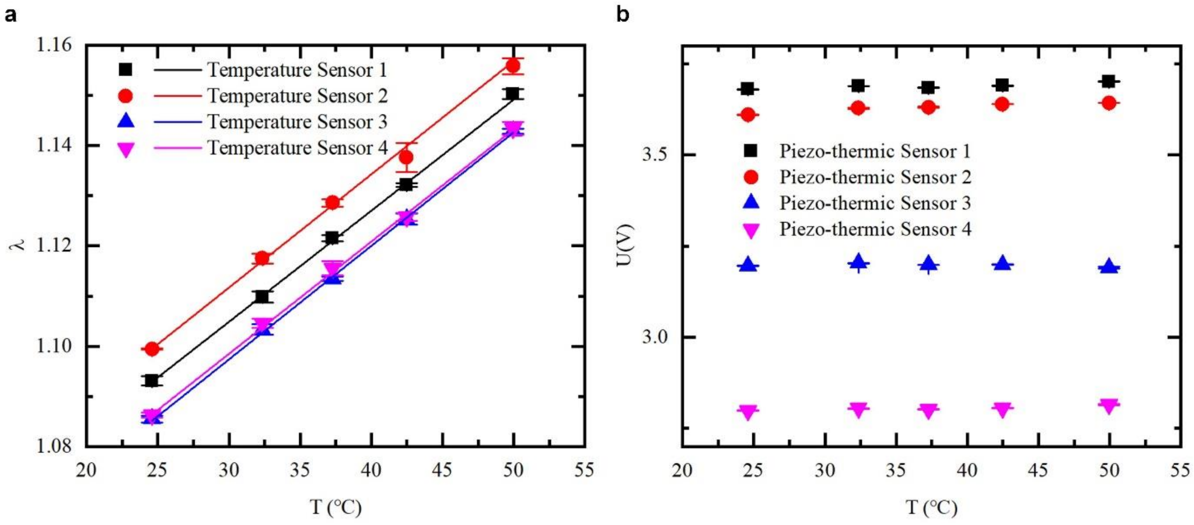

Figure S2. Responses of the piezo-thermic sensors on the e-skin to temperature, and temperature compensation results. a) Temperature responses of the temperature sensors (cold films) integrated on the piezo-thermic sensors. $\lambda = \frac{U_c}{U - U_c}$ is denoted as the output of the temperature sensor (cold film). Detail in Methods. The results show that the sensitivity of the temperature sensor in temperature sensing reaches about 0.0022 °C^{-1}. b) Temperature effect on the piezo-thermic sensor. The piezo-thermic sensors of e-skin are immune from the temperature variation.

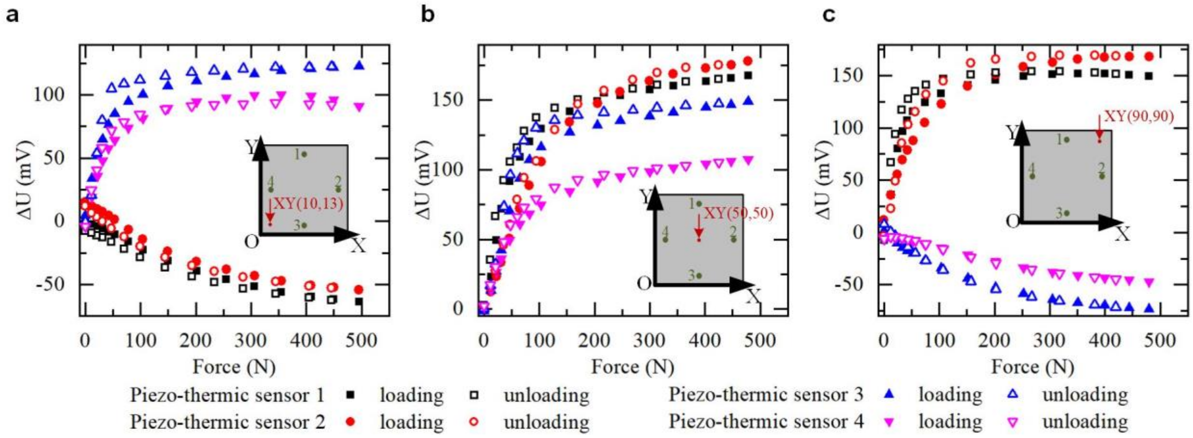

Figure S3. An example set of the responses of the e-skin to different forces and contact positions. An example set of the responses of the distributed piezo-thermic sensors on the e-skin when the forces ranging from 0~500 N are exerted on different contact positions on the e-skin. The insets show the layout of the contact positions (red arrows with XY coordinate) and the distributed piezo-thermic sensors (green points with serial numbers).

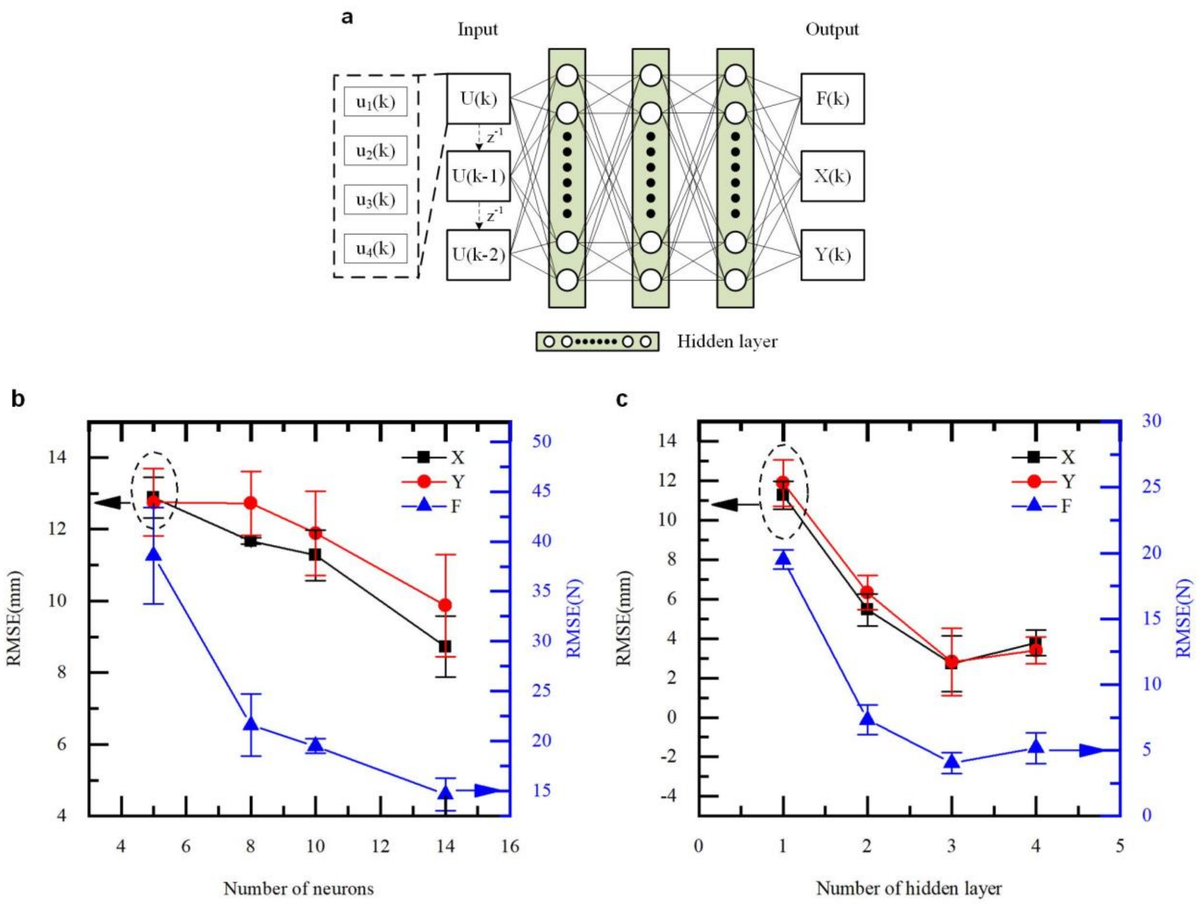

Figure S4. Network optimization. a) The schematic of the neural network structure. The time sequences of the outputs of distributed piezo-thermic sensors are used as the inputs of the network considering a second order prediction system, denoted as $U(k)$, $U(k-1)$, $U(k-2)$, while the applied force F and contact position coordinates (X, Y) are the outputs of the network. b) The RMSEs of the predicated force and position from the neural network with different numbers of neurons. The RMSEs of the network decrease with the increase of the neuron number and gradually saturate when the number of neurons is greater than 10. The error bars represent standard deviations from three runs. c) The RMSEs of the predicated force and position from the network with different numbers of hidden layers. The RMSEs of the predicated force and position reach the minimum when the number of hidden layers is 3. The error bars represent standard deviations from three runs. Therefore, the optimized neural network contains three hidden layers, and each hidden layer has 10 neurons.

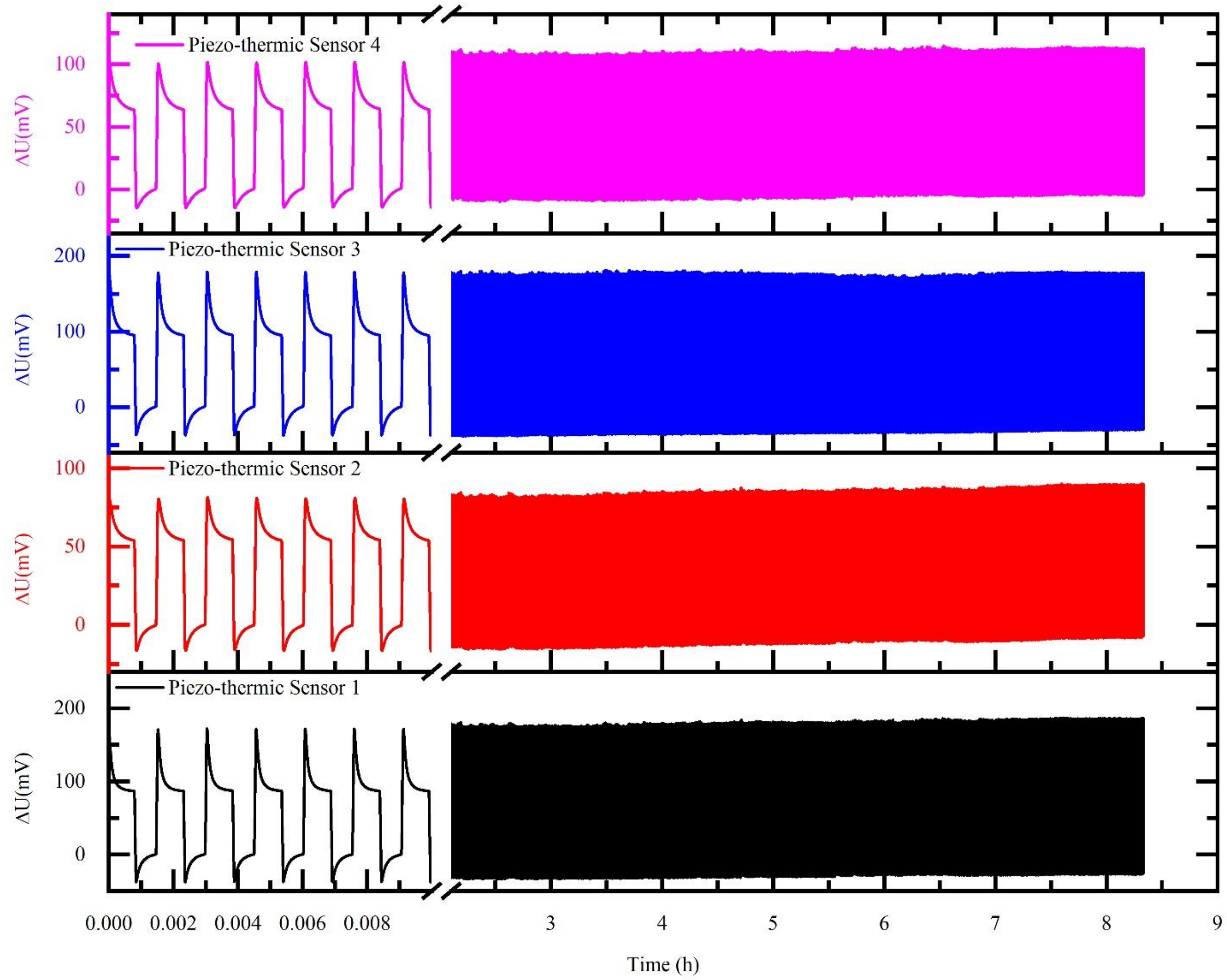

Figure S5. Collision repeatability and durability test on the e-skin. The force responses of the e-skin to periodical loading-unloading alternately changing between 0 and 50 N for 5000 cycles. The results indicate that the e-skin has good durability and stability in collision detection.

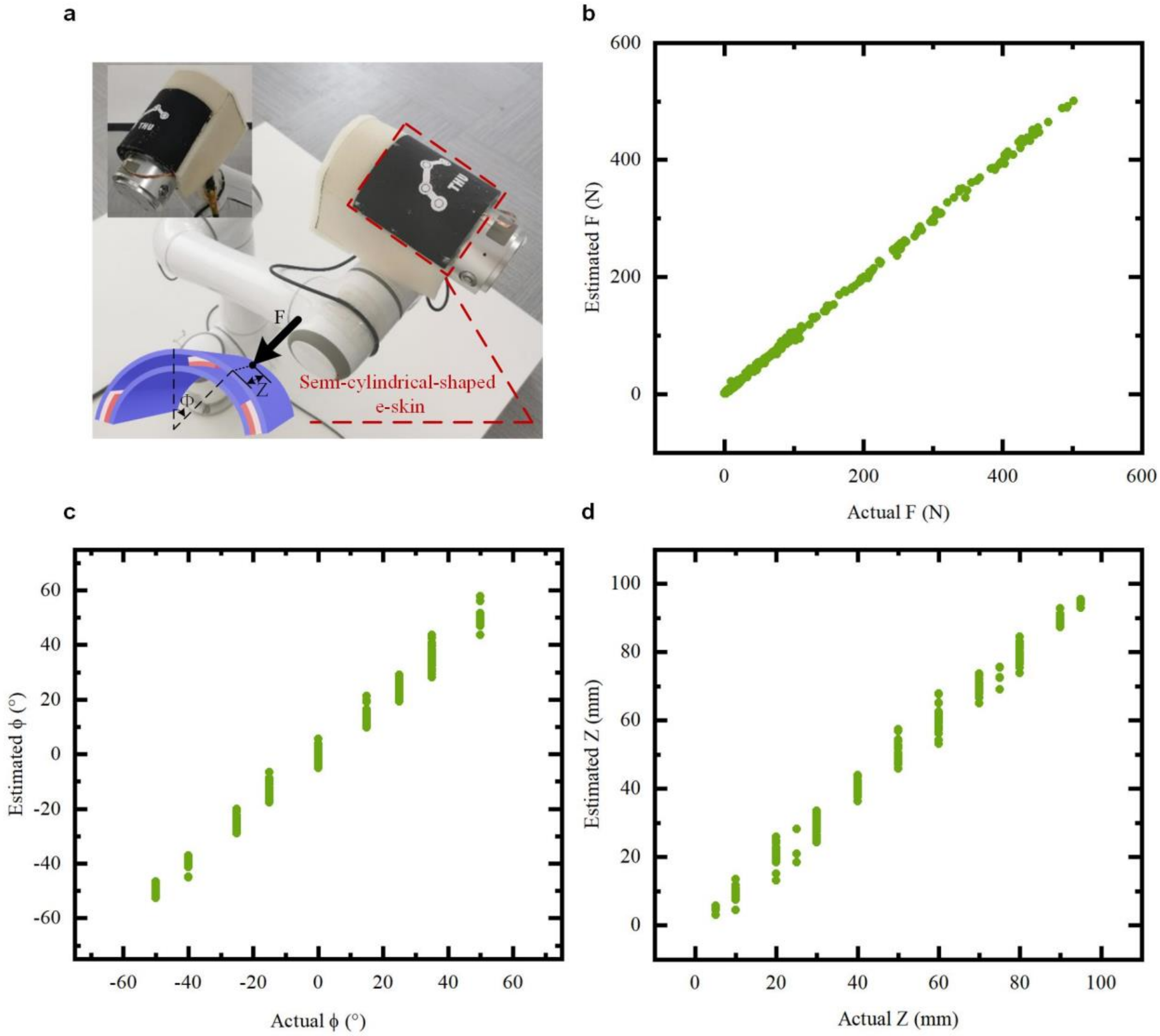

Figure S6. A semi-cylindrical-shaped e-skin and its performance for detecting contact force and contact position. a) A semi-cylindrical-shaped e-skin with an area of 13.9×10 cm^2 mounted on a cylindrical robotic arm. The inset shows that the contact position is expressed by cylindrical coordinates (i.e. central angle ϕ and Z). b) Measurement results of applied forces in the range of 0~500 N on the cylindrical-shaped e-skin. The RMSE of the force measurement is less than 3.5 N in the range of 0~500 N. c-d) Measurement results of the contact positions (ϕ , Z). The RMSEs of the contact position measurement reach 2.5 ^{o} in the range of -50 ^{o} ~50 ^{o} (Central angle) and 2.4 mm in the axial range of 0~100 mm (Z location).

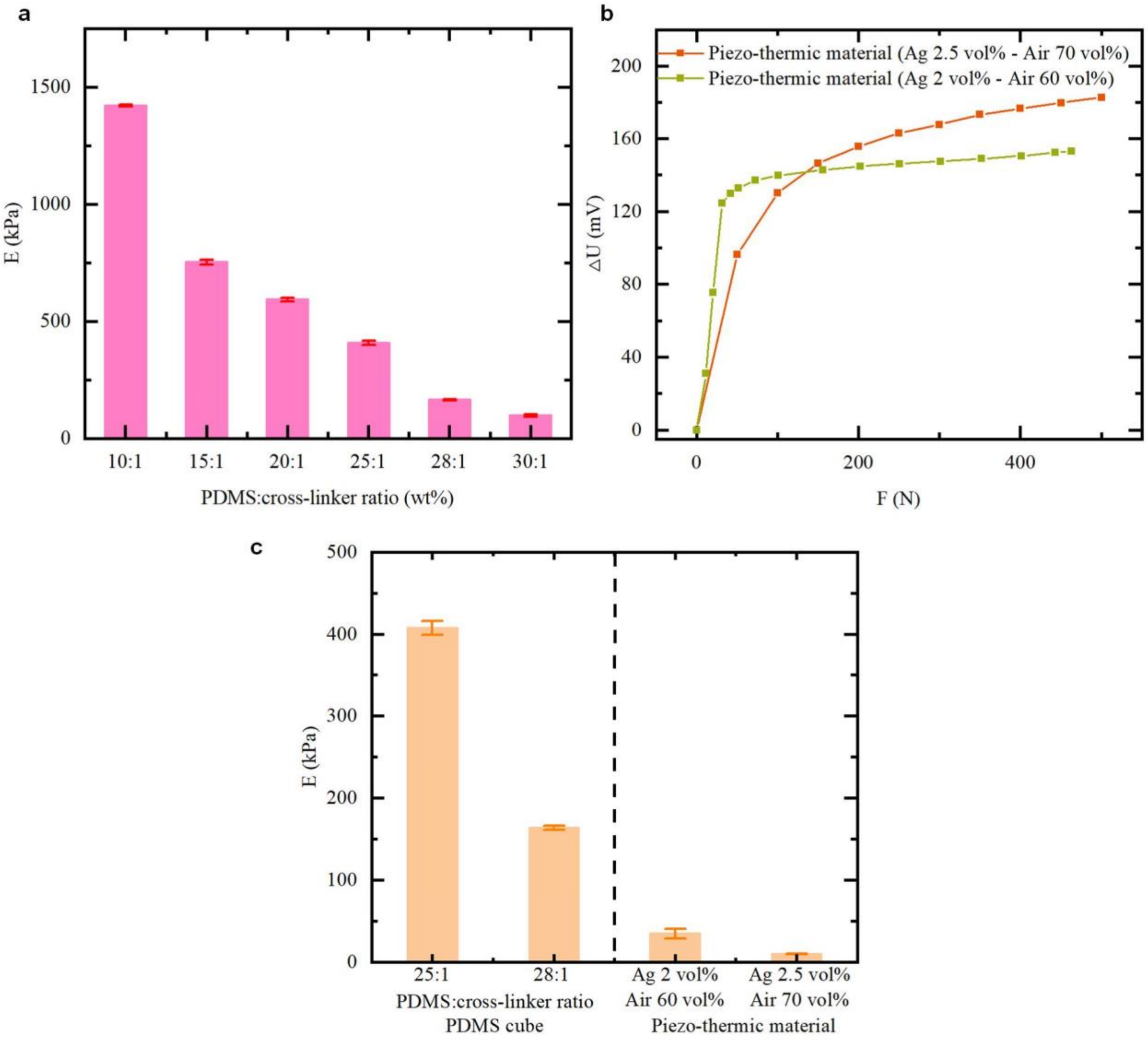

Figure S7. Optimization of force perception performance of the e-skin. a) The Young's moduli of the PDMS cubes with different cross-linker ratios. The optimized PDMS cube is composed of two PDMS layers with Young's moduli of 163 kPa (cross-linker ratio 28:1 wt%) and 407 kPa (cross-linker ratio 25:1 wt%) respectively. b) The experimental sensing performance of the thermal sensor paired with different porous materials. The results show that increasing the porosity and the ratio of Ag can improve the measuring range of the piezo-thermic sensor. The porosity and the ratio of Ag are optimized to be 70 vol% and 2.5 vol% respectively. c) Young's Moduli of the PDMS cubes and the porous materials with different compositions.

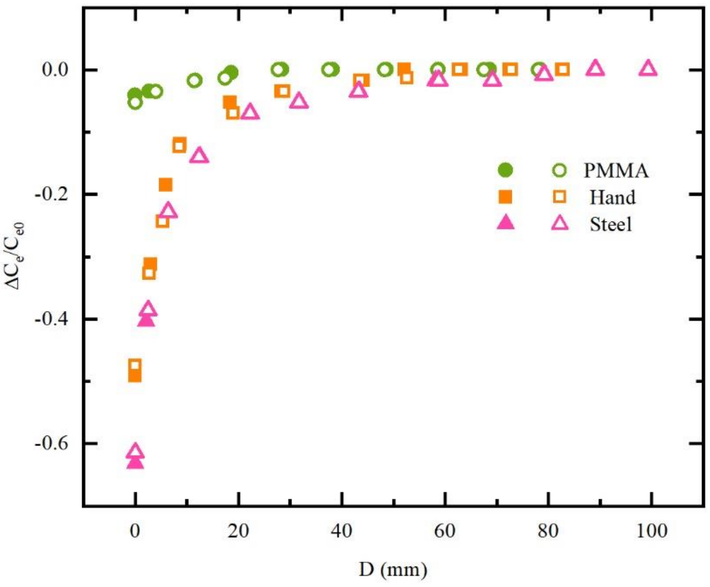

Figure S8. Responses of the e-skin to different objects approaching. Approaching run, filled symbols; moving away run, open symbols.

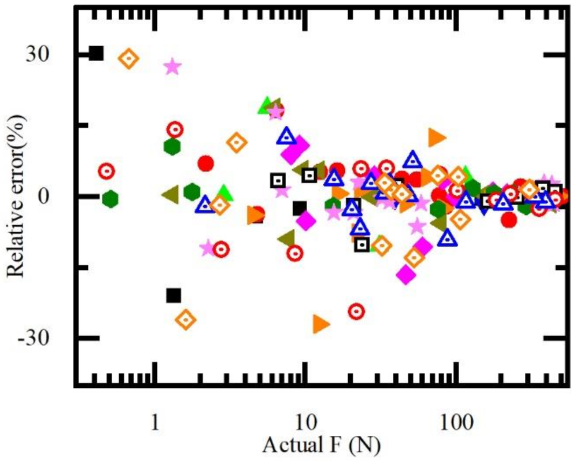

Figure S9. The relative errors of forces corresponding to results in Figure 1d-i.

List of Supplementary Files and Movies

Movie S1.
Summary of the multifunctional e-skin for application of robot collision avoidance, dexterous interaction with humans.

Movie S2.
The e-skin detects force and contact position in real-time when a finger touches it.

Movie S3.
The homemade robotic arm realizes collision avoidance and safety control by using the proximity sensing of the e-skin.

Movie S4.
The homemade robotic arm realizes collision detection and safety control by using the force sensing of the e-skin.

Movie S5.
The homemade robotic arm realizes proximity detection, collision detection and safety control by combining multi-sensation of the e-skin.

Movie S6.
The homemade robotic arm with the multifunctional e-skin interacts with humans and learns typing Morse code.

Movie S7.
The 6-DoF robotic arm realizes obstacle avoidance by using the proximity sensing of the e-skin.

Movie S8.
The 6-DoF robotic arm with the multifunctional e-skin plays Tai-Chi with a human.

Movie S9.
The 6-DoF robotic arm with the multifunctional e-skin interacts with humans, and learns handwriting the words ‘CHN’.
